# Sintered and 3D-Printed Bulks of MgB_2_-Based Materials with Antimicrobial Properties

**DOI:** 10.3390/molecules26196045

**Published:** 2021-10-06

**Authors:** Petre Badica, Nicolae Dan Batalu, Mariana Carmen Chifiriuc, Mihail Burdusel, Mihai Alexandru Grigoroscuta, Gheorghe Virgil Aldica, Iuliana Pasuk, Andrei Kuncser, Marcela Popa, Angelo Agostino, Lorenza Operti, Santanu Kumar Padhi, Valentina Bonino, Marco Truccato

**Affiliations:** 1 National Institute of Materials Physics, 405A Atomistilor Street, 077125 Magurele, Romania; mihaita_burdusel@yahoo.com (M.B.); alex_bebe07@yahoo.com (M.A.G.); aldica2000@yahoo.com (G.V.A.); iuliana.pasuk@infim.ro (I.P.); akuncser@yahoo.com (A.K.); 2 Faculty of Material Science and Engineering, University Politehnica of Bucharest, 313 Splaiul Independentei, 060042 Bucharest, Romania; dan_batalu@yahoo.com; 3 Faculty of Biology and The Research Institute of the University of Bucharest (ICUB), University of Bucharest, 91-95 Splaiul Independentei, 050095 Bucharest, Romania; carmen.chifiriuc@gmail.com; 4 Physics and Chemistry Departments, University of Turin, 1-7 Via Pietro Giuria, 10125 Turin, Italy; angelo.agostino@unito.it (A.A.); lorenza.operti@unito.it (L.O.); santanukumar.padhi@unito.it (S.K.P.); valentina.bonino@unito.it (V.B.); marco.truccato@unito.it (M.T.); 5 European Synchrotron Radiation Facility, 71 Avenue des Martyrs, 38000 Grenoble, France

**Keywords:** MgB_2_, antimicrobial activity, spark plasma sintering, machinable material, 3D printing

## Abstract

Pristine high-density bulk disks of MgB_2_ with added hexagonal BN (10 wt.%) were prepared using spark plasma sintering. The BN-added samples are machinable by chipping them into desired geometries. Complex shapes of different sizes can also be obtained by the 3D printing of polylactic acid filaments embedded with MgB_2_ powder particles (10 wt.%). Our present work aims to assess antimicrobial activity quantified as viable cells (CFU/mL) vs. time of sintered and 3D-printed materials. In vitro antimicrobial tests were performed against the bacterial strains *Escherichia coli* ATCC 25922, *Pseudomonas aeruginosa* ATCC 27853, *Staphylococcus aureus* ATCC 25923, *Enterococcus faecium* DSM 13590, and *Enterococcus faecalis* ATCC 29212; and the yeast strain *Candida parapsilosis* ATCC 22019. The antimicrobial effects were found to depend on the tested samples and microbes, with *E. faecium* being the most resistant and *E. coli* the most susceptible.

## 1. Introduction

Planktonic and biofilm-forming microbes are among the most important threats to human health. In the EU, 25,000 people die every year due to infections with antibiotic-resistant bacteria, and the management of these infections costs about 1.5 billion EUR/year [1]. This problem could be considered a crisis, because the rate of development and commercialization of novel effective antibiotics has slowed [2]. Moreover, government funds and efforts have recently been focused on other urgent problems, such as the COVID-19 pandemic crisis. From 1930 to 1962, 20 new types of antibiotics were developed; meanwhile, from 1962 to the present, only 2 new types of antibiotics have gone into production [3,4,5]. Modern antimicrobial strategies are needed [6], and among them, nanostructured materials such as powders, coatings, and bulks are promising candidates [7,8,9]. The literature also offers examples of many bioactive metals including Ag, Cu, Zn, Mg, Ce, Ti, Al, Si, Au, Bi, Ca, Fe, Pt, Sn, Hg, Cd, Cr, Tl, Al, Co, In, Ni, Mn, and Cr [10,11,12,13,14]. The first two, Ag and Cu [15], are the most popular, being already used in many antimicrobial applications. Metals are often used as oxides, hydroxides, halides, and sulfates [10,16] or they are introduced as active components in alloys (e.g., brasses, bronzes, copper–nickel–zinc) and in composite materials (e.g., steels, hydroxyapatite, polymer/resin matrices, and textiles [15,17]). Antimicrobial non-metals such as C (e.g., fullerene, carbon nanotubes, graphene oxide) [18,19,20,21,22] or B [23,24] and their compounds have also been reported to display antimicrobial functions. Other antimicrobial materials include quaternary ammonium compounds and synthetic or natural polymers (e.g., peptides, lactoferrin, chitosan) [11,15]. However, synthetic polymers often need physical, chemical, or mechanical surface modifications [15] to optimize their efficiency against microbes.

Magnesium diboride (MgB_2_) is well-known for its superconducting properties [25]. MgB_2_ can be prepared by a variety of methods, including spark plasma sintering (SPS) [26,27,28,29,30,31]. Our research group has recently reported for the first time in the scientific literature the potential for the antimicrobial applications of MgB_2_ materials (powders and biodegradable coatings based on polyvinylpyrrolidone) for the clinical field and for combating the negative impact of microbial colonization in different environments, as with the biodeterioration of heritage buildings [26,32,33,34,35]. We have previously shown that the antimicrobial activity of MgB_2_ powders depends on the fabrication process (e.g., MgB_2_ powders produced by reactive liquid infiltration (RLI) show superior performance to commercial powders) as well as their purity, microstructure, and pH behavior in water. Another important aspect revealed by our results was the good performance of these materials against a large spectrum of bacterial and fungal strains and their similar efficiency against microbes, both in the planktonic and the biofilm growth state. Biofilms are known to be significantly more resilient than individual microbes, and thus pose a higher health threat [36].

Considering the acute need for developing novel solutions to prevent and combat biofilms and the promising results reported in our previous papers for the antibiofilm potential of MgB_2_ coatings, we have continued our research in this direction. In the present paper, we focus on the bulk of this material to assess its in vitro antimicrobial activity against Gram-negative and Gram-positive bacteria, as well as yeast strains.

The bulk materials investigated in this work are sintered MgB_2_ high-density massive samples obtained by SPS and 3D printed samples of a polylactic acid (PLA) with the addition of MgB_2_ powder. For our study, we selected PLA for its wide availability, low price, biodegradability, and readiness for 3D printing [37,38,39]. PLA has great biocompatibility [39], and some of its applications are already in use (bone fixation screws, stent coating, bio-resorbable suture threads, etc.). The advantage of using the 3D-printing approach is that it allows the fabrication of functional medical devices with antimicrobial properties in diverse and complex shapes at low costs, and it is adapted for small series production. Bulks of sintered MgB_2_ were shown to have mechanical properties close to conventional structural SiC or Al_2_O_3_ ceramic (i.e., they are relatively hard and brittle) [40]. This makes it difficult to fabricate pristine MgB_2_ into precise and complex geometries by mechanical processing. Therefore, machinable MgB_2_ [41] sintered bulks with added hexagonal BN and 3D-printed materials with embedded MgB_2_ can provide the needed (complex) shape and size for applications.

The bulk materials based on MgB_2_ in this work that allow processing into complex shapes are envisioned for applications such as medical devices (artificial prosthetics and biodegradable implants [42,43], drug delivery systems) and self-sterilizing medical instruments with time- and space-controlled activity. Apart from the degradation and release of the active components with antimicrobial activity, the proposed bulks are also expected to allow mechanical support control. Other possible applications could be found in the packaging industry [44], such as for food transportation, preservation, and the enhancement of shelf life complimented by eco-friendly packaging with high levels of water biodegradability and biocompatibility. Other applications of bulk bioactive materials based on MgB_2_ could be found in industries where free surfaces of biological materials are necessary. The management of potable water could become a viable application [24], but other biofouling applications are also expected to emerge.

## 2. Materials and Methods

### 2.1. Bulks Fabrication

**Figure 1:**
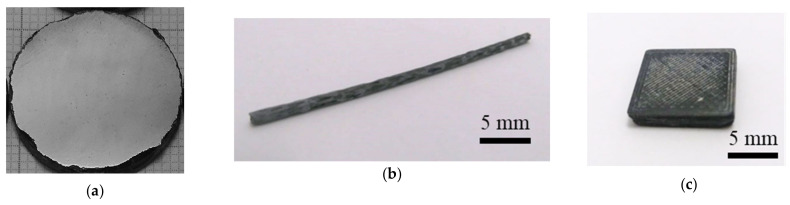
(**a**) MgB_2_ sintered metallographically polished disc; (**b**) filament of PLA with embedded MgB_2_ particles (10 wt.%) used for fabrication of the (**c**) 3D-printed sample.

High-density (>95%, Table 1) bulk samples of MgB_2_ (Figure 1a) and MgB_2_-added with 10 wt.% hexagonal BN (hBN) were prepared by SPS in a vacuum at 1150 °C for 3 min, under a maximum uniaxial pressure of 95 MPa. The raw powders were MgB_2_ (LTS Research Laboratories Inc, 99.5% purity, <44 µm) and hBN (High Purity Chemicals, >99%, 10 µm). The details of sample fabrication by SPS were reported in [41,45]. The hBN-added MgB_2_ was demonstrated to be machinable by chipping in [41].

Composite filaments of PLA (Figure 1b) with embedded MgB_2_ particles (LTS Research Laboratories Inc, Orangeburg, NY, USA, 99.5% purity, <44 µm) were prepared in two steps. In the first step, commercial PLA was dissolved in chloroform and mixed with MgB_2_ powder (10 wt.%). In the second step, after the evaporation of chloroform, the PLA+MgB_2_ solid layer was cut into small pellets and extruded as filaments [46] with a Noztek extruder. The composite polymer–ceramic filaments had an average diameter of 1.65 mm (±0.05). Printing into square shapes (~10 mm × 10 mm) with a thickness of ~3 mm (Figure 1c) was performed with a WASP 2040 Turbo 2 3D printer (based on fused filament fabrication, FFF) with a 0.7 mm nozzle diameter. The printing temperature was 210 °C and the bed temperature was 60 °C, with 100% infill and a printing speed of 20 mm/s.

### 2.2. Antimicrobial Assays

The antimicrobial activity of the MgB_2_-hBN sintered bulk and PLA-MgB_2_ 3D-printed samples was tested by using reference bacterial and fungal strains: *Escherichia coli* ATCC 25922, *Pseudomonas aeruginosa* ATCC 27853, *Staphylococcus aureus* ATCC 25923, *Enterococcus faecium* DSM 13590, *Enterococcus faecalis* ATCC 29212, and *Candida parapsilosis* ATCC 22019. 

The MgB_2_ bulk disks were incubated in a humid atmosphere at 37 °C for 6 and 24 h, in contact with microbial suspensions of 10^5^ CFU (colony-forming units)/mL density. After incubation, the materials were washed with distilled water to remove unadhered microorganisms and sonicated for 15 s at maximum power. Next, they were vortexed for 15 s at 3000 rpm to recover the adherent microorganisms that were quantified by determining the viable cells expressed as CFU/mL.

The antimicrobial activity of the bulk sintered MgB_2_-hBN was tested using a final inoculum density of 5 × 10^5^ CFU/mL prepared in a 10 mL saline solution, with the microbial viability being assessed after different contact times (0.5 h, 1 h, 2 h, 3 h, 4 h, 5 h, 6 h, 24 h) and expressed as CFU/mL. The antibacterial activity of PLA samples with embedded MgB_2_ particles was assessed by the direct contact method: A ~10^8^ CFU/mL microbial suspension was distributed over the sterile material samples and incubated in a humid atmosphere for 2 h, 4 h, and 24 h at 37 °C. After incubation, the colonized samples were placed in 2 mL of sterile saline and were vigorously shaken to detach the adherent bacterial cells. The harvested bacterial suspension was further used to prepare serial dilutions to quantify bacterial growth by counting the resulting colonies and calculating the colony-forming units (CFU/mL).

All the assays were performed in duplicate, and the statistical analysis of the obtained materials was performed using a paired *t* test with GraphPad Prism (version 8.0.0 for Windows, GraphPad Software, San Diego, CA, USA, www.graphpad.com, accessed on 4 October 2020).

### 2.3. Sample Characterization before and after Antimicrobial Test

The raw MgB_2_ powder and sintered bulk samples were subjected to X-ray diffraction (Bruker AXS D8 Advance diffractometer, CuKα radiation). Rietveld analysis (MAUD 2.31 [47]) was applied to determine the weight fraction of the phases, *a* and *c* lattice parameters of MgB_2_, the crystallite size, and the residual strain for different phases (Table 1). The amount of carbon (denoted *y*, Table 1) substituting boron in the crystal lattice of MgB_2_ (Mg(B_1−*y*_C*_y_*)_2_) was calculated with the empirical formula:

*y* = −21.9·*a* + 6.76 (*a* in nm) (1)

considering mediated data from [48,49,50].

The apparent bulk density of the sintered samples (r_a_) was measured by the Archimedes method. The relative density, R = (r_a_/r_t_) × 100 (%), where r_t_ is the theoretical mass density [51], was calculated by considering all identified phases (MgB_2_, MgO, MgB_4_, and Mg) as determined by Rietveld analysis [47]. The microstructure of the samples was observed using scanning electron microscopy (SEM, Lyra 3XMU/Tescan). Metallographic polishing of the sintered samples was made with different oil-based emulsions of Al_2_O_3_ down to a particle size of 1 µm. Saline suspensions with cultures (see Section 2.2) were placed on the as-polished surfaces.

## 3. Results and Discussion

We obtained, characterized, and bio-assessed the anti-biofilm activity of various types of samples based on MgB_2_ fabricated by SPS (pristine MgB_2_ and hBN-added MgB_2_) and FFF 3D printing. The obtained samples exhibited significant antimicrobial activity against adherent strains at short incubation times (less than 6 h) and against subsequently formed biofilms at longer incubation times of 24 h. This behavior represents an important advantage: the early occurrence of an inhibitory effect on bacterial growth prevents the development of bacterial biofilms on the surface of medical devices. The antimicrobial activity of MgB_2_, its good biodegradability and biocompatibility, as well as its anti-inflammatory properties promote this material as a useful candidate for a wide range of biomedical applications.

**Figure 2:**
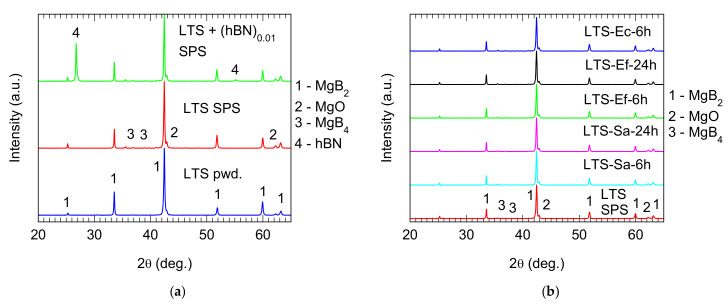
XRD patterns of (**a**) MgB_2_ raw powder (LTS), MgB_2_ as-sintered pristine (LTS SPS), and hBN-added (LTS + (hBN)_0.1_) MgB_2_ discs; (**b**) MgB_2_ pristine sintered discs before and after being in contact with different bacterial cultures (*Staphylococcus aureus* (SA), *Enterococcus faecium* (Ef), and *Escherichia coli* (Ec)) for different amounts of time.

XRD spectra and the results of Rietveld analysis on as-sintered pristine MgB_2_ samples are presented in Figure 2a and Table 1. The MgB_2_ raw powder was 97 wt.% MgB_2_ phase, and the impurity phases were MgO and metallic Mg (Table 1). After sintering, the MgB_2_ phase decreased by about 10 wt.% in the pristine sample, and the newly formed secondary phases were MgB_4_ and MgO. In the hBN-added sample, the amount of MgB_2_ was 78 wt.%. If ~10 wt.% of hBN is not taken into consideration, the maximum amount in the added sample would be ~88 wt.%, which is comparable to the 87.5% value in the pristine sample. As previously reported, hBN has little influence, if any, on the decomposition reactions of MgB_2_ during SPS [45]. The crystallite size of MgB_2_ during SPS showed some increase, but it was within the range of experimental error. The crystallite sizes of MgB_2_, MgO, and MgB_4_ for pristine and hBN-added SPS-processed samples can be considered similar. This result also supports the inertness of hBN compared to MgB_2_. In addition, there was little difference in the carbon intake during SPS between the pristine and hBN-added samples. As presented in Section 2.3, insertion of carbon in the crystal lattice of MgB_2_ decreased the *a*-axis lattice parameter, while the *c*-axis lattice parameter remained almost constant (Table 1). In addition, this is usually accompanied by an increase in microstrain. This trend can be observed when raw powder and sintered samples are compared. However, in the sintered samples, this correlation did not hold up, since the higher *y*-carbon level in sample LTS SPS (0.0114) than in the sample LTS + (hBN)_0.01_ SPS (0.0076) induced a lower microstrain of 0.12% vs. 0.14%, respectively. The amount of carbon, a biocompatible material, was very low in the sintered samples [52].

**Figure 3:**
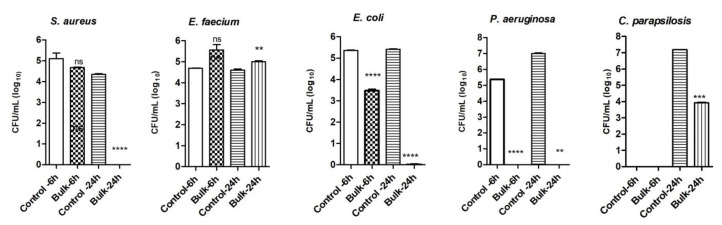
The number of viable microbial cells in log_10_(CFU/mL) for pristine MgB_2_ bulk sintered samples. Notations: ns = *P* > 0.05; * = *P* ≤ 0.05; ** = *P* ≤ 0.01; *** = *P* ≤ 0.001; **** = *P* ≤ 0.0001.

**Figure 4:**
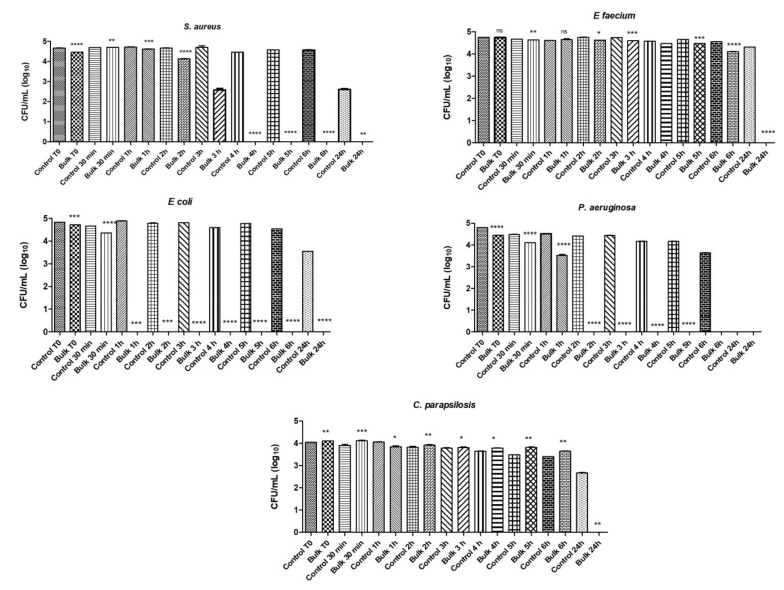
The number of viable microbial cells in log_10_(CFU/mL) for hBN-added MgB_2_ bulk sintered samples. Notations: ns = *P* > 0.05; * = *P* ≤ 0.05; ** = *P* ≤ 0.01; *** = *P* ≤ 0.001; **** = *P* ≤ 0.0001.

**Figure 5:**
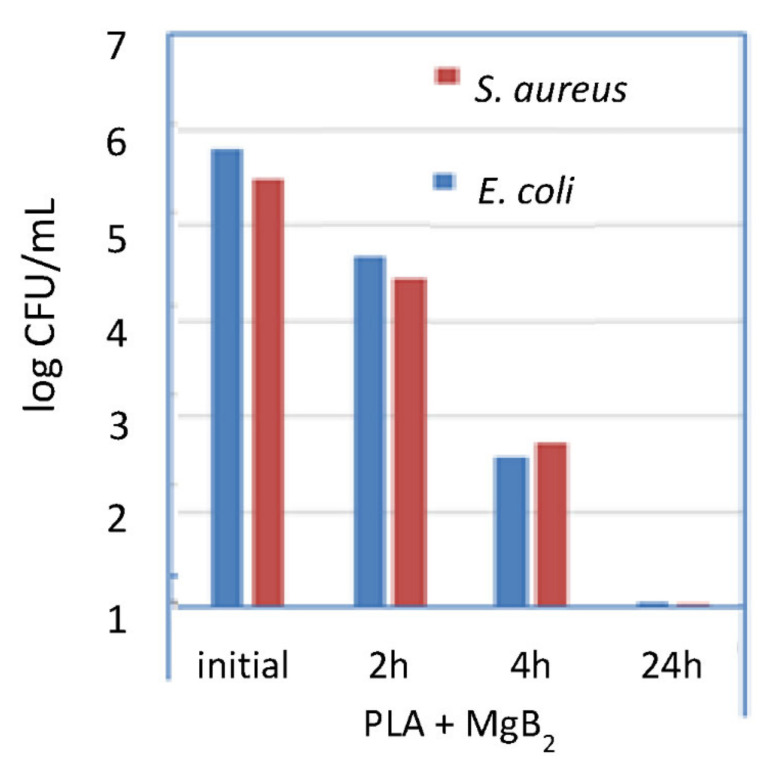
The number of viable microbial cells in log_10_(CFU/mL) for 3D-printed PLA+10 wt.% MgB_2_ samples.

The results of in vitro antimicrobial activity are presented in Figure 3, Figure 4 and Figure 5. The results demonstrate significant antimicrobial activity for the pristine MgB_2_ bulk material. Both the initial phase of adhesion of microorganisms quantified after 6 h of contact and the mature biofilm growth quantified after 24 h of incubation were inhibited. For pristine MgB_2_ sintered bulks, after 6 h of incubation there was a significant decrease in microbial growth for *E. coli* and *S. aureus* (Figure 3) and a total growth inhibition of the *P. aeruginosa* strain. After 24 h of incubation, the samples had completely inhibited the growth of four out of the five tested microbial strains (i.e., *E. coli*, *P. aeruginosa*, *S. aureus,* and *C. parapsilosis*). These results demonstrate significant antimicrobial activity of the pristine MgB_2_ bulk material. The initial phase of the microorganisms’ adhesion was quantified after 6 h of contact (statistically significant for *E. coli* and *P. aeruginosa*, *P* < 0.0005), and the mature biofilms’ growth was quantified after 24 h of contact.

The results for pristine MgB_2_ bulks were reproduced in machinable hBN-added MgB_2_ sintered samples (Figure 4). The results for the hBN-added samples showed that MgB_2_ had inhibitory and microbicidal effects on the microbial strains included in the study. Its efficacy was more pronounced for the Gram-negative strains *E. coli* and *P. aeruginosa*, which were no longer viable after 1 and 2 h of contact, respectively. Regarding the Gram-positive bacteria, the inhibitory effect of MgB_2_ against *S. aureus* started after 3 h of incubation and against *E. faecalis* after 6 h, and it was complete after 4 h and 24 h of incubation for *S. aureus* and *E. faecalis*, respectively. *C. parapsilosis* fungal strains are among the most frequently isolated fungi on human skin [53], and in our study the tested strain was the most resistant to the inhibitory effects of the tested samples. However, the fungicidal effect was initiated at 24 h. No viable cells of *P. aeruginosa* survived after 2 h of incubation. The comparison of the antimicrobial activity of the two types of sintered materials (i.e., pristine MgB_2_ bulk and hBN-added MgB_2_ sintered samples) reveals a higher efficacy of the latter against *E. faecium* after 6 h and 24 h of incubation, and similar activity against the other four tested microbial strains for those two incubation periods.

The 3D-printed PLA+10 wt.% MgB_2_ samples were tested for their anti-biofilm activity against two strains: the Gram-negative *E. coli* and the Gram-positive *S. aureus* (Figure 5). Statistical analysis was not performed for the polymer–MgB_2_ results. These results are preliminary, and more experiments are needed. The pristine PLA polymer is inert with respect to microbes [54], and therefore, if there is an antimicrobial effect it could likely be ascribed to the MgB_2_ from the 3D-printed samples. Analysis of the biofilm dynamics evidenced that the 3D-printed samples had an inhibitory effect on microbial cell adhesion after 2 h of incubation. At 24 h of incubation, the number of viable bacteria cells decreased by 5 log units, and no viable cells were observed at 48 h of incubation. These results suggest that it is necessary to carefully select the type and concentration of the composite components to improve and control antimicrobial activity over space and time.

**Figure 6:**
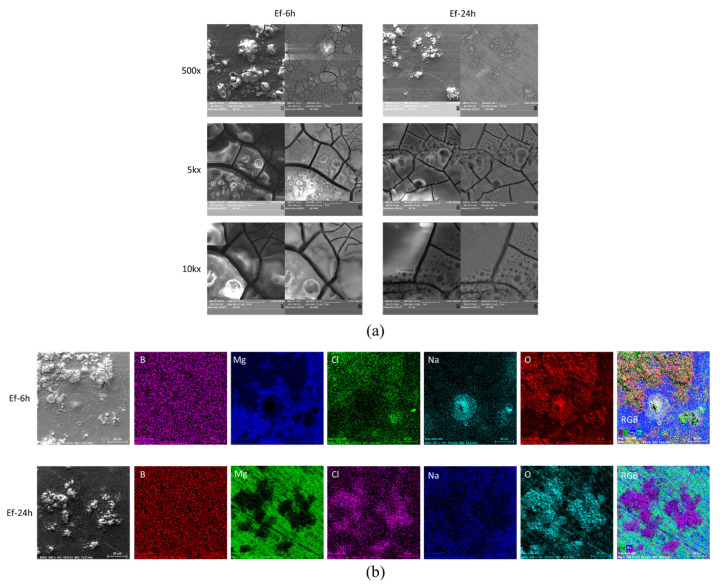
(**a**) SEM images (secondary electrons SE and backscattering BSE regimes), (**b**) BSE images, EDS elemental maps of MgB_2_ sintered bulk sample (LTS SPS, Table 1) after being in contact with *E. faecium*, and RGB images obtained by overlapping the EDS maps. The presence of Cl and Na on the surface is from the saline solution used for the in vitro tests.

The antimicrobial activity efficiency over time for a given material depends on the microbial strain. This raises the question of whether the adherent strains affect the surface structure of the colonized material over time. If this were the case, it could partially explain the differences observed in the intensity of the antimicrobial effect exhibited by different surfaces in contact with different strains for different contact times. Figure 2b shows the XRD spectra of the surface of the pristine MgB_2_ sintered bulk samples after removing the *E. coli*, *E. faecium*, and *S. aureus* biofilms. There are no notable differences among the samples. A similar result can be inferred from the microscopy data (Figure 6), where, to avoid redundancy, only the samples that were in contact with *E. faecium* are shown. When surfaces of samples in contact with other strains were investigated (not shown here) no particularities were revealed.

We conclude that the process of surface corrosion develops independently of the microbial strain, and it could be influenced by the saline solution. Corrosion-specific features are similar to those reported in [35]. More research is needed on corrosion vs. the antimicrobial effect of MgB_2_-based materials.

Taken together, the biological assays suggest significant antimicrobial activity of MgB_2_ materials (pristine, added, or composite), making them promising candidates for the development of novel antimicrobial strategies. Further studies are required to establish the detailed mechanisms of the antimicrobial activity revealed by the tested MgB_2_-based materials. However, we could speculate that the antimicrobial activity may be due to the release of Mg^2+^ ions, which could affect the integrity of microbial cell membranes or disrupt membrane potential and cause the leakage of cellular contents and eventually cell lysis [54,55,56,57,58]. The anti-biofilm effect of Mg^2+^ ions could result from downregulation of extracellular matrix gene expression [59] or from the enhancement of c-di-GMP degradation, which would decrease biofilm formation [60]. Moreover, considering the anti-inflammatory effects of magnesium, we expect that the use of these materials in antimicrobial formulations could lead to the attenuation of tissue lesions caused by an increased inflammatory response to the presence of pathogens [61].

## 4. Conclusions

In this paper, we outlined how we obtained, characterized, and bio-evaluated the anti-biofilm activity of samples based on MgB_2_ fabricated by SPS (pristine MgB_2_ and hBN-added MgB_2_) and as 3D-printed composite (PLA embedded with 10 wt.% MgB_2_ particles). These samples inhibited both the initial phases of biofilm development quantified after 6 h of incubation and the mature biofilms at 24 h. The antimicrobial activity of MgB_2_, its good biodegradability and biocompatibility, as well as its anti-inflammatory properties promote this material as a useful candidate for a wide range of biomedical applications, including the development of novel biomaterials resistant to microbial colonization that would present a low risk for developing medical device biofilm-associated infections.

## Figures and Tables

**Table 1:** Samples (discs), apparent and relative densities, lattice constants *a* and *c* of MgB_2_, the level of carbon substitution *y* in Mg(B_1−*y*_C*_y_*)_2_, the residual strain of MgB_2_, phases content, and average crystalline size. Phases identified in XRD patterns of bulk MgB_2_: MgB_2_ (ICDD 38-1369), MgB_4_ (ICDD 73-1014), and MgO (ICDD 45-0946), Mg (ICDD 35–0821), and hBN (ICDD 34-0421).

Sample	Apparent Density, (g/cm^3^)/Relative Density, (%)	MgB_2_ Lattice Parameter, *a*, (Å)		MgB_2_ Lattice Parameter, *c*, (Å)	Amount of Carbon *y* in Mg (B_1−*y*_C*_y_*)_2_		Micro-Strain of MgB_2_ (%)	
LTS pwdr.	-	3.0863 ± 0.0001		3.5221 ± 0.0001	0.0011 ± 0.0003		0.075	
LTS SPS	2.61/99.3	3.0821 ± 0.0002		3.5253 ± 0.0001	0.0114 ± 0.0006		0.12 ± 0.04	
LTS + (hBN)_0.01_ SPS	2.53/95.0	3.0840 ± 0.0002		3.5271 ± 0.0001	0.0076 ± 0.0005		0.14 ± 0.08	
**Sample**	**Phase amount (wt.%)**				**The average crystallite size from XRD (nm)**			
	MgB_2_	MgB_4_	MgO	Mg/hBN	MgB_2_	MgB_4_	MgO	Mg/hBN
LTS pwdr.	97 ± 0.5	0	1.8 ± 0.2	1.2 ± 0.1/-	113 ± 5	-	45 ± 2	51 ± 30/-
LTS SPS	87.5 ± 0.6	3.9 ± 0.1	8.6 ± 0.1	-/-	130 ± 15	105 ± 20	50 ± 6	-
LTS + (hBN)_0.01_ SPS	78.0 ± 0.4	3.6 ± 0.1	7.9 ± 0.2	-/10.5 ± 0.2	153 ± 14	140 ± 65	50 ± 8	-/79 ± 19

## Data Availability

The raw/processed data required to reproduce these findings cannot be shared at this time as the data also form part of an ongoing study. Data are available from the corresponding authors on request.
